# Supplementary material for: Comprehensive Landscape of RRM2 with Immune Infiltration in Pan-Cancer
Source: Cancers (Basel). 2022 Jun 14;14(12):2938. doi: 10.3390/cancers14122938 (PMC9221307; doi:10.3390/cancers14122938)
Supplement: Supplementary file 1 [file cancers-14-02938-s001.zip › cancers-1745370-supplementary.pdf]

Supplementary Materials: Comprehensive Landscape of RRM2 with Immune Infiltration in Pan-cancer

Zijian Zhou, Qiang Song, Yuanyuan Yang, Lujia Wang and Zhong Wu

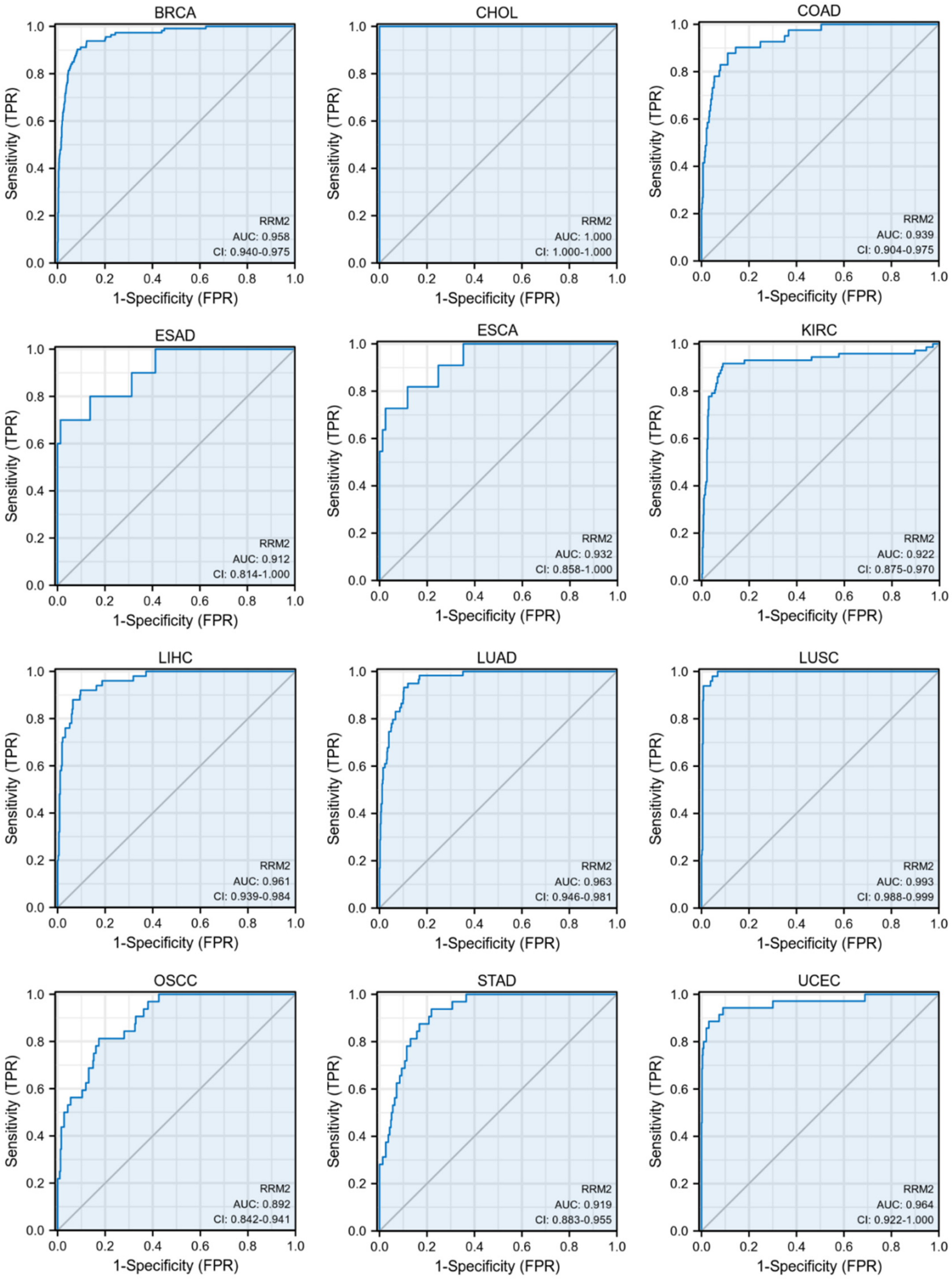

Figure S1. The prediction accuracy of RRM2 in pan-cancer by ROC analysis.

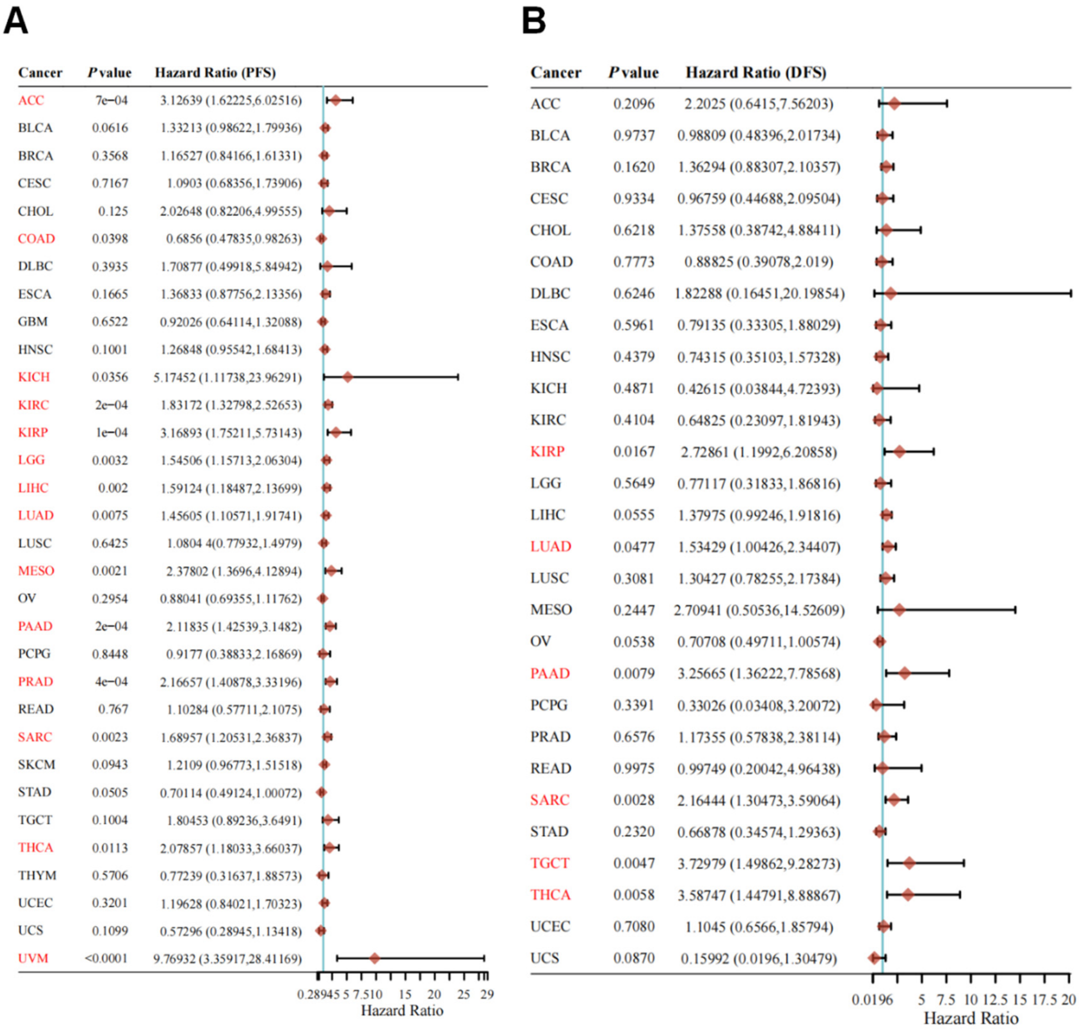

Figure S2. Relationship of RRM2 expression with PFS and DFS. (A-B) Forest map shows the univariate Cox regression analysis results of PFS (A) and DFS (B) for RRM2 in TCGA pan-cancer samples.

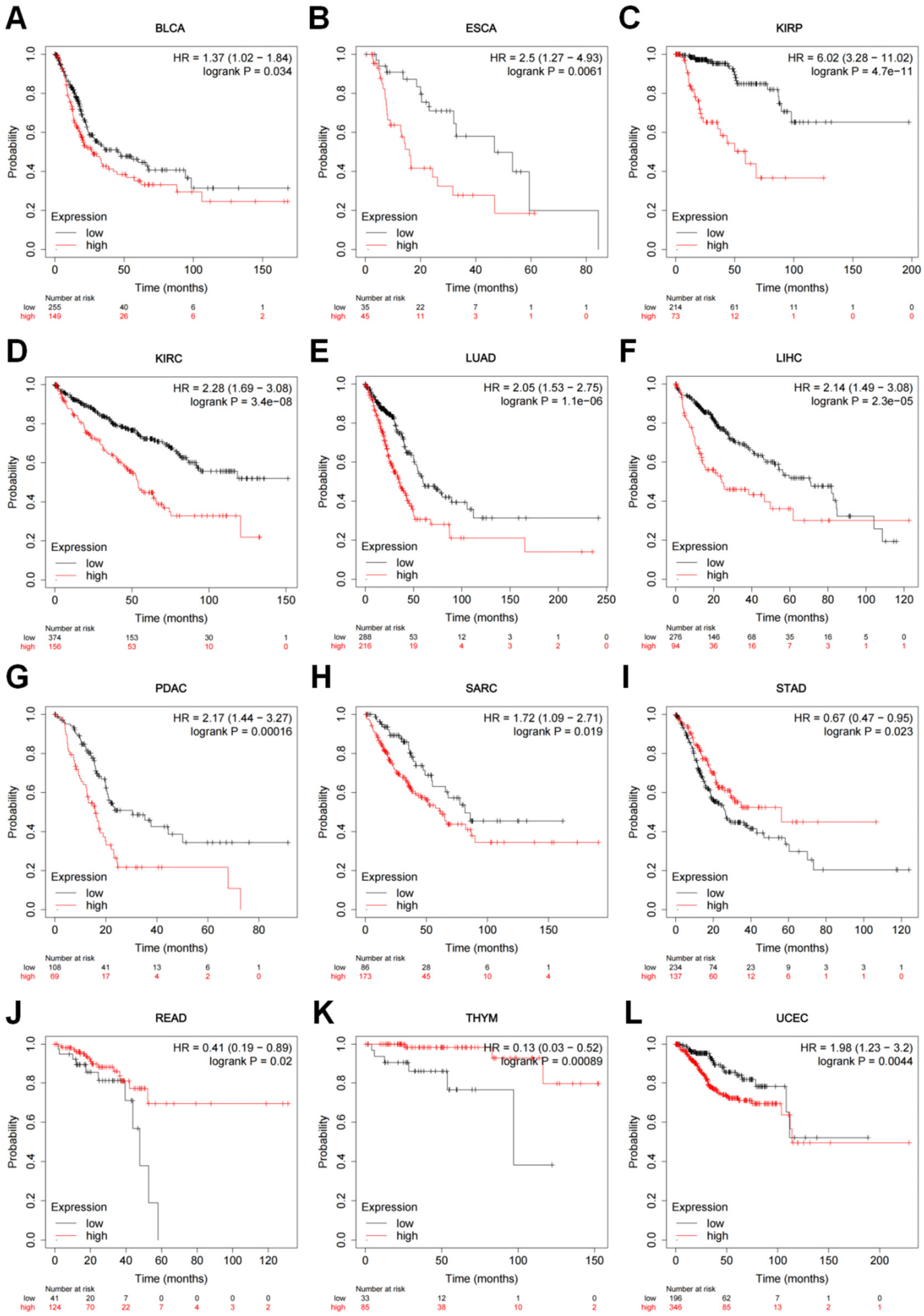

Figure S3. OS curves based on low and high expression of RRM2 in pan-cancer via K-M plotter.

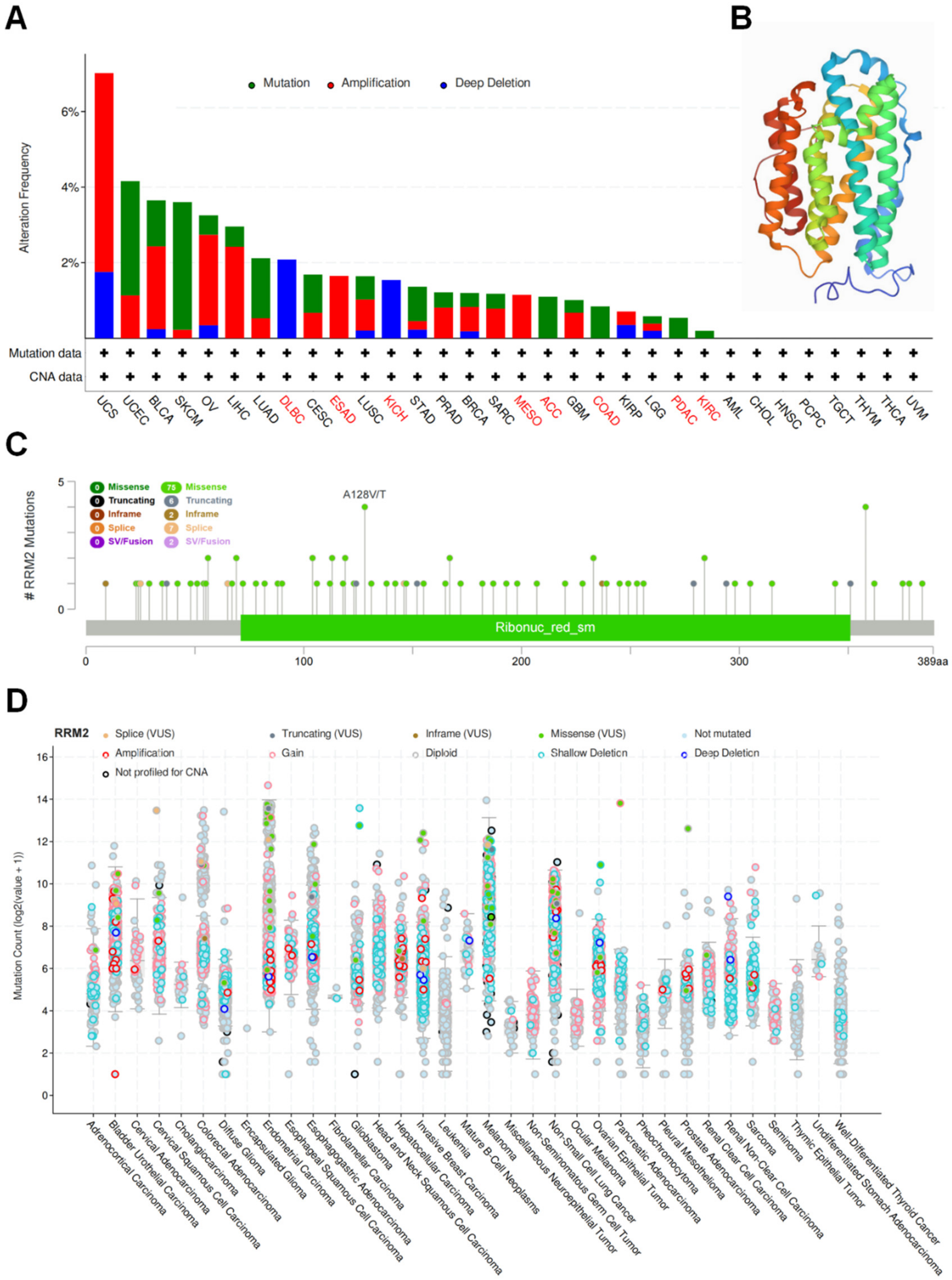

Figure S4. Mutation landscape of RRM2 in cancers with cBioPortal database. (A) RRM2 genetic alteration type and frequency in TCGA with Ciopo. (B) The 3D structure of RRM2. (C-D) The general mutation sites (C) and mutation counts (D) of RRM2 in in various cancers.

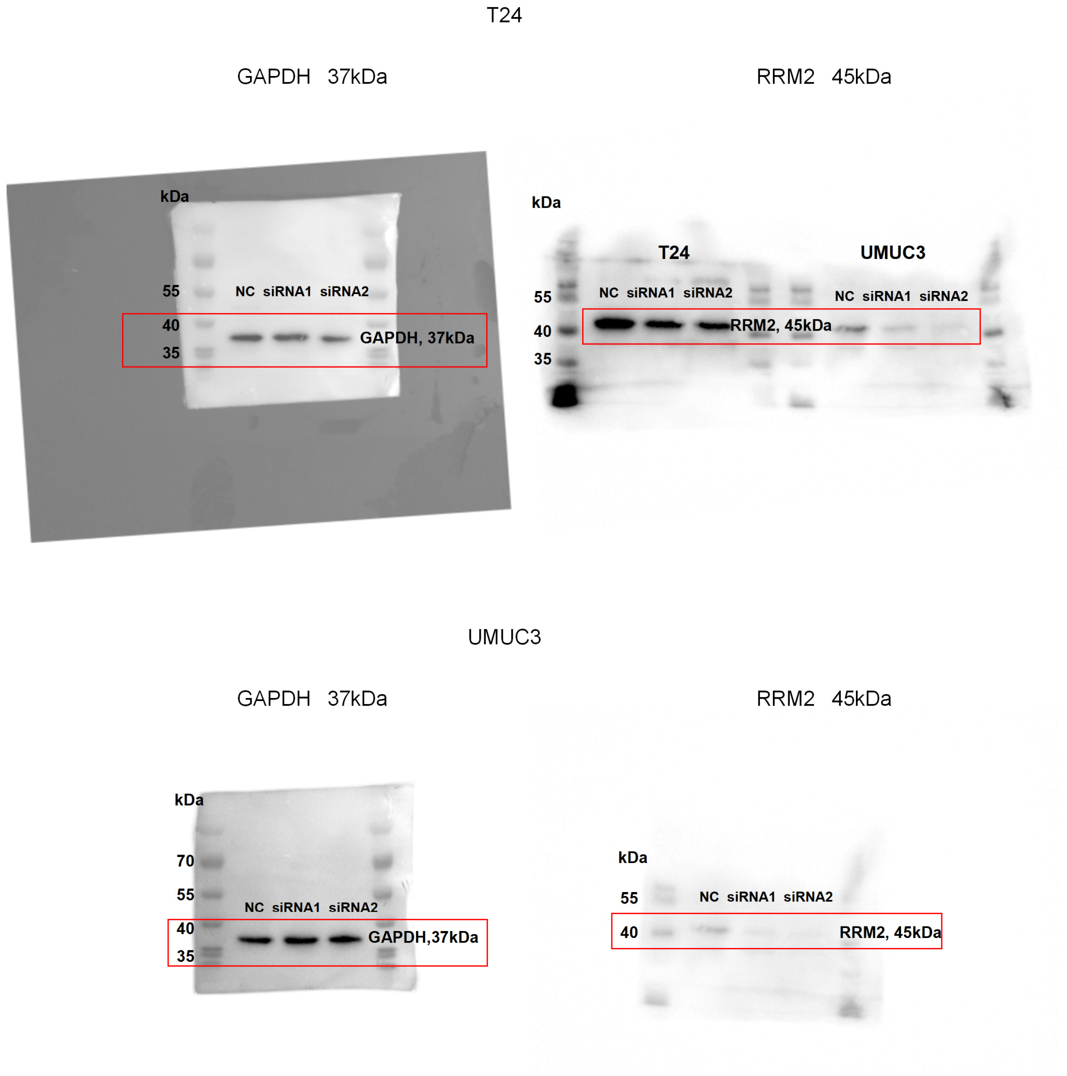

Figure S5. The original uncropped western blots.

Table S1. Main reagents and consumable used in this article.

Reagents	Vendor	Catalogue Number	Information
Antibody-RRM2	ProteinTech	11661-1-AP	WB: 1:1000
Antibody-GAPDH	ProteinTech	60004-1-Ig	WB: 1:5000
Primer-RRM2	Tsingke Biotech-nology	/	Forward: 5′-GTGGAGCGATTTAGCCAAGAA-3′<br>Reverse: 5′-CACAAGGCATCGTTTCAATGG-3′
Primer-Actin	Tsingke Biotech-nology	/	Forward: 5′- ATGACTTAGTTGCGTTACACC -3′<br>Reverse: 5′- GACTTCCTGTAACAACGCATC -3
siRNA-RRM2	Hanbio Biotech-nology	HH20220208RFF-SI01	**siRNA-NC** Sence: UUCUCCGAACGUGUCACGUTT<br>Anti-Sence: ACGUGACACGUUCGGAGAATT<br>**siRNA-RRM2-1** Sence: GGAUGUGCUGCAAGACGUAGATT<br>Anti-Sence: UACGUCUUGCAGCACAUCCCATT<br>**siRNA-RRM2-2** Sence: GAACAUAGGUCUUGGAAUAAATT<br>Anti-Sence: UAUUCCAAGACCUAUGUUCUGTT
Lipofectamine 3000	Invitrogen	PH0429	Carlsbad, CA, USA

Table S2. Correlation analysis between RRM2 and markers of immune cells in BLCA via TIMER.

Description	Gene markers	KIRC			
		None		Purity	
		Cor	*P*	Cor	*P*
**B cell**	CD19	-0.046	0.354	-0.048	0.356
	CD79A	-0.032	0.522	-0.043	0.405
**T cell (general)**	CD3D	0.065	0.189	0.033	0.528
	CD3E	0.120	*	0.101	0.053
	CD2	0.136	**	0.116	*
**CD8+ T cell**	CD8A	0.236	***	0.224	***
	CD8B	0.187	***	0.170	**
**Monocyte**	CD86	0.239	***	0.225	***
	CD14	0.183	***	0.166	**
	CSF1R	0.134	**	0.114	*
**TAM**	CD11b	0.178	***	0.172	**
	CD68	0.252	***	0.242	***
	IL10	0.135	**	0.144	**
**M1 Macrophage**	IRF5	-0.104	*	-0.106	*
	PTGS2	0.067	0.175	0.069	0.187
	CD40	-0.105	*	-0.127	*
**M2 Macrophage**	MRC1	0.206	***	0.199	***
	CD163	0.226	***	0.214	***
	VSIG4	0.202	***	0.191	***
	CD200R1	0.126	*	0.107	*
	MS4A4A	0.179	***	0.176	**
**Neutrophils**	CEACAM8	0.064	0.199	0.065	0.215
	CCR7	-0.228	***	-0.240	***
**Natural killer cell**	KIR3DL1	0.086	0.082	0.055	0.289
	KIR3DL2	0.236	***	0.229	***
	KIR3DL3	0.183	***	0.169	**
**Dendritic cell**	HLA-DPB1	0.135	**	0.124	*
	HLA-DRA	0.200	***	0.188	***
	NRP1	0.306	***	0.314	***
	ITGAX	0.172	***	0.162	**

*$P<0.05$, **$P<0.01$, ***$P<0.001$.
